# Advancements in Research on Genetic Kidney Diseases Using Human-Induced Pluripotent Stem Cell-Derived Kidney Organoids

**DOI:** 10.3390/cells13141190

**Published:** 2024-07-13

**Authors:** Do Hyun Na, Sheng Cui, Xianying Fang, Hanbi Lee, Sang Hun Eum, Yoo Jin Shin, Sun Woo Lim, Chul Woo Yang, Byung Ha Chung

**Affiliations:** 1 Transplantation Research Center, College of Medicine, The Catholic University of Korea, Seoul 06591, Republic of Korea; dhrah@catholic.ac.kr (D.H.N.); cuishengmd@catholic.ac.kr (S.C.); xianyingfang@catholic.ac.kr (X.F.); hanbilee89@gmail.com (H.L.); trickyspot@gmail.com (S.H.E.); dbwls612@gmail.com (Y.J.S.); swlim@catholic.ac.kr (S.W.L.); yangch@catholic.ac.kr (C.W.Y.); 2 Division of Nephrology, Department of Internal Medicine, Seoul St. Mary’s Hospital, The College of Medicine, The Catholic University of Korea, Seoul 06591, Republic of Korea; 3 Division of Nephrology, Department of Internal Medicine, Incheon St. Mary’s Hospital, The College of Medicine, The Catholic University of Korea, Incheon 21431, Republic of Korea

**Keywords:** human-induced pluripotent stem cell (hiPSC), kidney organoid, genetic kidney disease, gene editing

## Abstract

Genetic or hereditary kidney disease stands as a pivotal cause of chronic kidney disease (CKD). The proliferation and widespread utilization of DNA testing in clinical settings have notably eased the diagnosis of genetic kidney diseases, which were once elusive but are now increasingly identified in cases previously deemed CKD of unknown etiology. However, despite these diagnostic strides, research into disease pathogenesis and novel drug development faces significant hurdles, chiefly due to the dearth of appropriate animal models and the challenges posed by limited patient cohorts in clinical studies. Conversely, the advent and utilization of human-induced pluripotent stem cells (hiPSCs) offer a promising avenue for genetic kidney disease research. Particularly, the development of hiPSC-derived kidney organoid systems presents a novel platform for investigating various forms of genetic kidney diseases. Moreover, the integration of the CRISPR/Cas9 technique into this system holds immense potential for efficient research on genetic kidney diseases. This review aims to explore the applications of in vitro kidney organoids generated from hiPSCs in the study of diverse genetic kidney diseases. Additionally, it will delve into the limitations of this research platform and outline future perspectives for advancing research in this crucial area.

## 1. Introduction

Chronic kidney disease (CKD) has been recognized as a major public health problem globally. It can significantly increase the incidence of cardiovascular complications and mortality, and greatly impair quality of life due to various complications. In the majority of CKD patients, the primary kidney diseases are diabetes mellitus (DM), hypertension (HTN) or glomerulonephritis [1,2]. However, genetic or hereditary kidney diseases also cannot be overlooked as a cause of CKD occurrence. Genetic kidney disease shows an overall prevalence of about 60~80 cases per 100,000 in Europe and the USA and 10–15% of adults and most pediatric patients who progress to renal-replacement therapy have hereditary kidney disease [3,4]. In Korea, according to a report by a Korean Society of Nephrology, hereditary kidney disease accounts for 2.4% of the causes of end-stage renal failure [5,6]. In addition, among the 12.5% of patients whose cause is unknown, it is estimated that a significant number are due to hereditary kidney disease [5,6].

The advancement of genomic research and widespread application of gene testing for diagnosing genetic diseases have unveiled the genetic causes of various kidney diseases, transforming many cases previously thought to be CKD of unknown origin into genetic kidney diseases. Furthermore, defects in various genes are continually being reported to be associated with CKD development [4,7,8]. Therefore, more than 600 genes have been implicated in monogenic kidney diseases, and known single-gene disorders account for up to 50% of nondiabetic CKD in pediatric cohorts and 30% in adult cohorts [4]. Despite these advancements, there is still no clear treatment that can suppress the progression of CKD in many genetic kidney diseases; hence, many patients progressed to end-stage renal disease requiring dialysis treatment [3]. There could be several reasons for this, but above all, the lack of appropriate animal models for studying the pathogenesis of diseases and developing new drugs, as well as the difficulty in conducting clinical trials due to a small number of patients, are likely to be the most important factors.

Meanwhile, over the past decade, kidney organoids generated from human-induced pluripotent stem cells (hiPSCs) have become innovative in vitro models for studying kidney disease [9,10,11,12,13]. Compared to animal or in vitro 2D culture cell models, the kidney organoids system has many advantages for the research of genetic kidney disease. For example, utilizing this system can address the limitations of animal models that may exhibit differences from human tissues, and furthermore, we can create more sophisticated 3D human kidney tissues, which cannot be observed in 2D cell cultures [14,15,16]. Thus, this review will discuss the research on modeling genetic kidney diseases using hiPSC-derived kidney organoids and their applications, and will also explore future directions in this field.

## 2. Human-Induced Pluripotent Stem Cells and Kidney Organoids System

Embryonic stem cells (ESCs), renowned for their capacity to proliferate indefinitely while retaining pluripotency and the capability to differentiate into cells of all three germ layers, have long served as a cornerstone in the research of various diseases and regenerative medicine [17]. However, ethical issues are the major obstacles for the use of ESCs in the research and this has prompted a search for solutions [18]. In 2006, when Takahashi and Yamanaka made the groundbreaking discovery that mouse skin fibroblasts could be reprogrammed into an induced pluripotent stem cell (iPSC) state sharing the ESC’s unlimited self-renewal and pluripotent differentiation capabilities, using a simple mixture of pluripotent transcription factors, the previously mentioned question was answered perfectly. A year later, the success in converting human fibroblasts into human-induced pluripotent stem cells (hiPSCs) made it feasible to acquire and perpetuate a practically limitless supply of healthy or disease-specific human pluripotent stem cells [19,20,21].

The process of nephrogenesis in vitro starts with the differentiation of hiPSCs into intermediate mesoderm (IM) cells, which then further differentiate into metanephric mesenchyme (MM) and ureteric bud (UB) cell populations. These two cell populations interact reciprocally to form the structures of the kidney. The MM gives rise to nephron progenitors (NPs), which eventually form the nephron segments, while the UB undergoes branching morphogenesis to form the collecting duct system [22,23,24]. The induction of kidney organoids typically involves a stepwise differentiation protocol, guided by specific growth factors and signaling molecules like Wnt, FGF and BMP to mimic the developmental stages of the kidney [25,26,27,28].

For instance, the protocols developed by Takasato et al. and Taguchi et al. focus on generating nephron-like structures, while other approaches, such as the one by Uchimura et al., combine separately induced MM and UB populations to improve the maturation and functionality of kidney organoids [29,30]. This modular approach allows for a more accurate recreation of the complex interactions and cell types present in the developing kidney, enhancing the physiological relevance of the resulting organoids.

With the advancement of hiPSC-related research, there is active progress in the production of organoids mimicking various organs from hiPSCs [31,32,33]. For kidney organoids, Taguchi and colleagues were the first to successfully differentiate hiPSCs into “kidney organoids”, namely multicellular systems containing podocytes, proximal tubules and distal tubules in a segmented structure arranged in a nephron-like unit [23]. Nowadays, some other research groups have also developed their own protocols for generating iPSC-derived kidney organoids, and research on applying these “mini-kidneys” to the research and regenerative therapy of kidney diseases is actively underway [22,24,26,27,28,29,34].

## 3. Applications of Kidney Organoids for Genetic Kidney Disease Modeling

**Figure 1:**
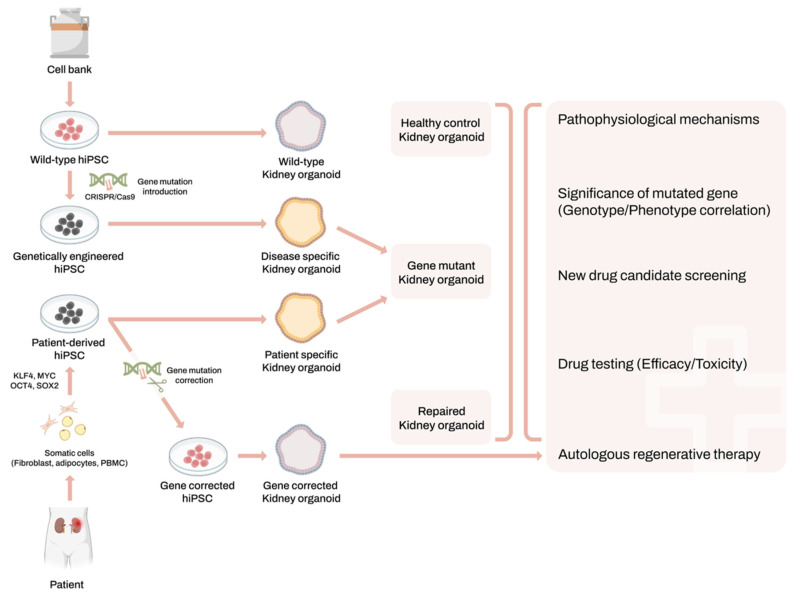
Scheme of process for the modeling of genetic kidney disease using hiPSC. * hiPSC; human-induced pluripotent stem cell.

The kidney organoid system is well-suited for studying the mechanisms of genetic kidney diseases, drug discovery and toxicology [35,36,37,38]. Mutant hiPSCs can be generated either by isolating somatic cells from patients with genetic kidney disease or by introducing disease-specific mutations into wild-type (WT) hiPSCs using CRISPR/Cas9 technology. Especially, CRISPR/Cas9 gene editing technology has significantly facilitated this area of research. Genome engineering has allowed for the generation of knockout lines, the correction of mutations in patient-derived hiPSCs, providing a large number of gene expression data that have been helpful in decoding the intricate mechanisms of human renal organogenesis [26]. Subsequently, disease-specific kidney organoids can be generated from these mutant hiPSCs. This approach enables the reproduction of genetic disease mechanisms and cell type heterogeneity within the kidney using hiPSC-derived kidney organoids [11,35,36,37] (Figure 1). Following Freedman’s successful integration of CRISPR/Cas9 technology into kidney organoid systems for modeling autosomal dominant polycystic kidney disease (ADPKD), several teams are now endeavoring to utilize similar platforms to model various diseases [26,35]. 

Significant advancements have been made in the use of kidney organoids derived from non-human mammalian iPSCs, which offer essential insights and complementary data to human studies. For example, Van Den Berg et al. showed that PSC-derived kidney organoids transplanted into mice can induce neo-vasculogenesis and significant glomerular and tubular maturation, demonstrating the potential of mouse models [39]. Additionally, kidneys have been generated from PSCs via blastocyst complementation in rodents, establishing functional renal structures in animal models [40,41].

Matsui et al. further demonstrated cross-species organoid research feasibility by injecting rat renal progenitor cells into neonatal mice, forming mature chimeric nephrons. They also generated human nephrons in neonatal mice, confirming the potential of human organoids in preclinical drug screening and pathology analysis [42]. These studies highlight the valuable contributions of rodent models in advancing kidney organoid research, providing critical insights into the development, integration and functional assessment of organoids, which are essential for translating findings into human applications.

In the subsequent paragraphs, we will introduce research findings related to diseases that have been modeled using human kidney organoid systems up to the present (Table 1).

### 3.1. Autosomal Dominant Polycystic Kidney Disease

ADPKD is the most common genetic kidney disease, predominantly caused by mutations in two genes: *PKD1* (Chr. 16.p13.3; approximately 78% of families) and *PKD2* (4p21; approximately 15%). Additionally, a rare third locus, *GANAB* (11q12.3; approximately 0.3%), was discovered in 2016 [59].

In 2015, Freedman and colleagues established their protocol for differentiating hiPSCs into nephron organoids, marking the creation of the first ADPKD kidney organoid model by knocking out the *PKD1* or *PKD2* genes in hiPSCs [26]. Leveraging this organoid system, they successfully developed a high-throughput screening platform for investigating ADPKD pathogenesis, as well as for toxicity and efficacy assessments in drug development [35]. Subsequently, in 2021, they generated kidney tubuloids using a distinct population of CD24+ renal epithelial cells possessing unique metabolic and gene regulatory programs, demonstrating that CD24+ cell-derived renal tubules can be utilized to establish an ADPKD model using multiplexed CRISPR-Cas9 gene editing, rapidly inducing cyst formation [60]. This highlights the potential of specific cell differentiation for improved disease modeling. By 2024, they had developed base-edited PKD organoids representing four common nonsense mutations. Heterozygous mutants exhibited no cyst formation, suggesting possible therapeutic avenues. They identified eukaryotic ribosomal selective glycosides (ERSGs) as PKD therapeutics, enabling the ribosomal readthrough of these same nonsense mutations [61].

In 2021, Yasaman Shamshirgaran and colleagues introduced a faster protocol for the direct differentiation of CRISPR-targeted cell pools, utilizing a doxycycline-inducible Cas9-expressing hiPSC line for high-efficiency editing instead of genetically modified clonal lines. They generated *PKD1* and *PKD2* mutant hiPSCs with >80% editing efficiency, differentiating them into kidney organoids with cystogenesis, providing a platform for rapid target validation in the context of disease modeling [44].

Kidney development entails complex interactions among nephron progenitors, forming renal tubules and glomeruli, and ureteric buds (UBs), which give rise to the collecting duct. Most studies have generated cysts from proximal and/or distal nephron tubules using nephron organoids. However, large cysts in ADPKD patients tend to originate from collecting ducts rather than nephron tubules. In 2020, Shohei Kuraoka and colleagues demonstrated cyst formation in ureteric bud organoids derived from iPSCs with homozygous deleted PKD1, as well as in ureteric bud organoids generated from heterozygous mutant iPSCs and from a patient with ADPKD, all upon cAMP stimulation. These UB organoids suggested that cyst formation is regulated by the balance between cAMP- and PC1/PC2-mediated signals [45]. As shown above, as organoid fabrication techniques develop and more sophisticated organoids become feasible, the intricate modeling of human ADPKD becomes possible.

Recently, genetic studies revealed a growing list of genes associated with PKD [62,63]. Liu et al. created hiPSCs with a homozygous knockout of *GANAB*, and forskolin-treated *GANAB*-/- kidney organoids showed significant tubular cyst formation, marked by aberrant cAMP metabolism and calcium homeostasis, similar to *PKD1* and *PKD2* mutations. This highlighted *GANAB*’s role in maintaining normal kidney tubule function. Targeting the cilium-autophagy signaling pathway, via genetic interventions like ATG5 overexpression or primary cilia ablation, and pharmacological activation of autophagy with FDA-approved minoxidil, significantly reduced cystogenesis, demonstrating the therapeutic potential of this pathway both in vitro and in vivo [46].

### 3.2. Autosomal Recessive Polycystic Kidney Disease

Autosomal recessive polycystic kidney disease (ARPKD) is a severe inherited cystic disease characterized by the combination of bilateral renal cystic disease and congenital hepatic fibrosis [64,65]. ARPKD manifests at birth or during childhood, and it is an important cause of pediatric morbidity and mortality. ARPKD is caused by mutations in Polycystic Kidney and Hepatic Disease 1 (*PKHD1*) or less commonly in DAZ interacting zinc finger protein 1 (*DZIP1L*). The *PKHD1* gene encodes fibrocystin (FPC) and leads to most of ARPKD, while the *DZIP1L* gene is associated with moderate ARPKD [63,66]. Although ARPKD shows similarities to ADPKD, with dysregulated ciliary pathways, proliferation, apoptosis and fluid secretion observed in both, they have distinct histopathological features and cellular characteristics [67].

In 2019, Low and colleagues generated hiPSCs from an ARPKD patient, and then used CRISPR/Cas9 to correct a specific mutation in the *PKHD1* gene. In ARPKD kidney organoids, elevated levels of intracellular cAMP triggered significant cyst formation in a dose-dependent manner. Conversely, corrected ARPKD organoids exhibited only minimal cyst formation, mirroring the phenotype observed in wild-type (WT) hiPSCs-derived kidney organoids [47]. They evaluated effects of thapsigargin and a CFTR inhibitor on forskolin-induced cystogenesis in ARPKD organoids, and both drugs prevented cyst growth in a dose-dependent manner, consistent with previous studies using human PKD cells and mouse models. These results affirm the potential of the kidney organoid platform as a physiologically relevant model for preclinical drug evaluation before clinical trials.

### 3.3. Fabry Disease Nephropathy

Fabry disease (FD) is a glycoshingolipid lysosomal storage disorder resulting from a deficiency in the α-galactosidase A (α-GalA) enzyme due to mutations in the *GLA* gene. It is characterized by the progressive intracellular accumulation of globotriaosylceramide (Gb3) in various types of cells including podocytes, renal tubular epithelial cells and vascular endothelial cells [68]. Despite advances in therapeutic technologies such as enzyme replacement therapy or chaperon therapies, the lack of humanized experimental models of Fabry disease nephropathy (FDN) has limited the development of new therapies to overcome the limitations of previously used therapies [69,70].

In 2021, Kim et al. demonstrated that hiPSC-derived *GLA*-mutant nephron organoids showed significant deformation of podocytes and tubular cells with the accumulation of Gb3, increased oxidative stress and apoptosis. Enzyme replacement treatment (ERT) with recombinant human α-Gal A decreased the Gb3 accumulation and oxidative stress, which resulted in the amelioration of the deformed cellular structure of the *GLA*-mutant kidney organoids. In addition, glutathione replacement treatment decreased oxidative stress and attenuated the structural deformity of the *GLA*-mutant kidney organoids [48].

In our previous studies, we used both patient-derived and also *GLA*-knock out hiPSCs by CRISPR/Cas9 for FDN modeling [49,69,70]. First, kidney organoids were generated from *GLA*-mutant hiPSCs derived from PBMCs of two male FD patients with different *GLA* mutations (classic and non-classic types). Compared to WT, Fabry patient-derived hiPSCs and kidney organoids exhibited decreased α-GalA activity and increased Gb3 deposition, particularly in classic-type mutations. Multi-lamellated inclusion bodies were observed in mutant kidney organoids but not in WT. This suggested that kidney organoids derived from male Fabry patients recapitulated the disease phenotype, reflecting the severity based on the *GLA* mutation type [49]. Our next aim was to investigate whether the CRISPR/Cas9-mediated suppression of *A4GALT*, the Gb3 synthase coding gene, could rescue the phenotype of FDN in *GLA* mutant kidney organoids. We generated FD-patient-derived hiPSC and *GLA*-KO hiPSCs and additionally performed *A4GALT*-KO in both *GLA*-mutant hiPSCs. Using these hiPSCs, we generated kidney organoids and compared FDN phenotypes. As a result, in both *GLA*-mutant-kidney organoids, α-GalA activity was significantly decreased along with the increased deposition of Gb3 in comparison with WT organoids. An intra-lysosomal inclusion body was also detected under EM. However, these FDN phenotypes were rescued by KO of *A4GALT* in both *GLA*-mutant-kidney organoids. Hence, it can be proposed as a therapeutic approach to treat FDN [69]. These research findings suggest that glutathione or *A4GALT* could serve as potential targets for novel FDN treatments, while also indicating the provision of an appropriate platform for the development of therapeutics for FDN.

### 3.4. Gitelman Syndrome

Gitelman’s disease (GIT) is a genetic tubular disorder with an autosomal recessive inheritance pattern. Mutations in the solute carrier family 12 member 3 (*SLC12A3*) gene, which encodes the sodium chloride cotransporter (NCCT) in distal convoluted tubule (DCT) [71], also known as the thiazide sensitive cotransporter, underlie this disease. In patients with GIT, less sodium is reabsorbed in the DCT due to defective NCCT, resulting in increased excretion of potassium, hydrogen and hypokalemic alkalosis [72]. This disorder currently lacks fundamental treatment options beyond conservative measures like potassium supplementation. To model GIT, we have generated hiPSC derived from a GIT patient [51]. In addition, we corrected the *SLC12A3* gene mutation by using the CRISPR-Cas9 system, and hence generated corrected hiPSCs. When we differentiated both mutant and corrected hiPSCs into nephron organoids, decreased NCCT mRNA levels and protein in *SLC12A3* mutant kidney organoids were observed in comparison with WT-organoids. However, they were normalized in the gene-corrected organoids [50]. This research successfully models GIT for the first time in terms of decreased expression of NCCT in E-CAD (+) cells within kidney organoids and demonstrates that gene correction can normalize it. However, we did not show the defective movement of electrolytes, which are directly related to the onset of this condition. It would be possible with the creation of more sophisticated organoids that encompass blood vessels, generate urine and reproduce their flow.

### 3.5. Karyomegalic Interstitial Nephritis

Karyomegalic interstitial nephritis (KIN) is a rare form of chronic interstitial nephritis first reported in 1974 [73]. KIN is a genetic kidney disease caused by mutations in the FANCD2/FANCI-Associated Nuclease 1 (*FAN1*) gene on 15q13.3, which results in karyomegaly and fibrosis of kidney cells through incomplete repair of DNA damage.

The first step we undertook for the modeling of KIN using the kidney organoid system was to generate hiPSCs derived from patients who were diagnosed with KIN after kidney biopsy and also had a mutation in the *FAN1* gene seen with DNA sequencing (CMC-KIN) [74]. In addition, we discarded the *FAN1* gene in WT hiPSCs (WTC-11) using the CRISPR/Cas9 system, and thus generated *FAN1*-edited hiPSCs (WTC-11 *FAN1*+/-). Using *FAN1*-mutant hiPSCs and also WT hiPSC, we generated kidney organoids and induced DNA damage by treating the kidney organoids with mitomycin C [52]. As a result, mitomycin C treatment significantly increased the expression of DNA damage markers in *FAN1*-mutant kidney organoids (CMC-KIN and WTC-11 *FAN1*+/-), but it not in WT-kidney organoids. These results suggest that *FAN1*-mutant kidney organoids can recapitulate the phenotype of KIN, and we expect that this can be a valuable platform for investigating the mechanisms by which CKD develops due to defective DNA repair.

### 3.6. Alport Syndrome

Alport syndrome (AS) is the second most common hereditary glomerulonephritis characterized by progressive glomerulosclerosis, leading to renal failure, as well as extrarenal complications. It is caused by mutations in COL4A3, COL4A4 or COL4A5 genes, affecting type IV collagen in the glomerular basement membrane (GBM) [75,76,77]. Despite the severity of the disease, there is no cure, and developing a preclinical platform that accurately recapitulates the disease phenotypes has been challenging.

Some research groups successfully modeled AS using hiPSCs and a nephron organoid system [53,54]. In 2023, Hirayama et al. generated hiPSCs from two male AS patients, differentiated these hiPSCs into kidney organoids and then evaluated these organoids for their ability to model AS. These organoids expressed altered type IV collagen α5 (IV), and those derived from hiPSCs with corrected *COL4A5* mutations restored collagen α5 (IV) protein expression. The model successfully recapitulated the phenotypic differences in collagen composition observed between mild and severe AS cases. Furthermore, the study demonstrated that treatment with the chemical chaperone 4-phenyl butyric acid (4-PBA) could correct GBM abnormalities in organoids with mild AS phenotypes by restoring α5(IV) protein expression. However, this treatment was not effective in severe AS organoids, highlighting the potential for personalized medicine approaches in AS treatment based on the specific genetic mutations present [53].

In another significant study, Morais et al. investigated the dynamics of basement membrane (BM) assembly, identifying key BM isoforms altered by a pathogenic COL4A5 variant and finding dynamic BM composition regulation from development to adulthood. Using iPSC lines from AS patient, they found that AS patient-derived organoids formed normal glomeruli and tubules by light microscopy but showed increased LAMB2 deposition, especially in extraglomerular BM. This study highlighted that kidney organoids could model abnormal BM assembly in human development and disease, aligning with glomerular laminin dysregulation seen in AS patients and animal models [54].

### 3.7. APOL1 Nephropathy

Genetic variants in the Apolipoprotein L1 (*APOL1*) gene are found only in African ancestry. The *APOL1* risk variants cause large increases in susceptibility to multiple different types of kidney disease including hypertension-associated ESRD, FSGS and HIV-associated nephropathy [78]. To date, there is no definitive evidence that the course of *APOL1*-mediated kidney disease is ameliorated by any particular regimen in CKD presentations [79].

In a study by Freedman lab, kidney organoids model of *APOL1* nephropathy were transcriptomically profiled [55]. They explored how these genetic variations influence the transcriptomic profiles of different kidney cell types, offering insights into the pathophysiology of nephropathy at a single-cell resolution. It is expected that this organoid model will provide a novel platform for studying the pathophysiology of *APOL1*-mediated kidney disease.

In another study, using CRISPR-Cas9, researchers developed human-derived *APOL1* G0/G0 and *APOL1* G2/G2 kidney organoids, finding that DGAT2 inhibition increased lipid droplets and reduced APOL1-mediated cytotoxicity in high-risk *APOL1* G2/G2 organoids [56]. This study suggests that modulating lipogenesis and lipid droplet formation through DGAT2 inhibition could be a potential therapeutic strategy for mitigating the cytotoxic effects of *APOL* 1 risk variants.

### 3.8. Autosomal Dominant Tubulointerstitial Kidney Disease (ADTKD)

ADTKD is the third most common monogenic kidney disease that affects the tubules and interstitial tissue, leading to progressive kidney failure. It is caused by mutation in five different genes (*UMOD*, *MUC1*, *REN*, *SEC61A1* and *HNF1β*) [80,81].

In their 2018 study, Aneta Przepiorski and her team used CRISPR/Cas9 to create a knockout of the *HNF1β* transcription factor gene in kidney organoids. The HNF1β-/- organoids exhibited decreased expression of markers linked to the proximal tubules (PTs) such as *LRP2* and to the thick ascending limb (TAL) including *UMOD* and *SLC12A1*. This suggests that the absence of HNF1β disrupts the normal development of PT and TAL regions in kidney organoids [82]. In another study, heterozygous KO (*HNF1β*+/-) ureteric bud organoids derived from hiPSCs were developed. *HNF1β*+/- organoids showed a loss of apical–basolateral polarity and had reduced numbers of budding regions [83]. Greka’s laboratory generated kidney organoids from *MUC1*-mutant patient iPSCs, demonstrating an intracellular accumulation of mutant MUC1 protein. In addition, they identified a small molecule (BRD4780) that cleared the mutant MUC1 protein in lysosomal degradation by using patient-derived kidney organoids [84].

### 3.9. Nephronophthisis (NPHP)

Nephronophthisis (NPHP) is an autosomal recessive, progressive tubulointerstitial kidney disease histologically characterized by the disruption of the tubular basement membrane, tubular dilatation and atrophy, interstitial fibrosis and decline to ESKD [85]. IFT14- plays an important role in the retrograde intraflagellar transport that is associated with NPHP. Forbes and his team generated organoids from a patient with *IFT140* mutation iPSCs and isogenic gene-corrected iPSCs. These organoids demonstrated shortened, club-shaped primary cilia and a polarization defect, whereas gene correction rescued this phenotype [58].

### 3.10. Autosomal Recessive Renal Tubular Dysgenesis (AR-RTD)

Autosomal Recessive Renal Tubular Dysgenesis (AR-RTD) is a fatal genetic disorder characterized by the complete absence or severe depletion of proximal tubules. It is caused by pathogenic variants in any one of four genes in the Renin–Angiotensin Aldosterone System (RAAS): angiotensin-converting enzyme (*ACE*), angiotensin ΙΙ receptor type 1 (*AGTR1*, coding for the AT1R protein), angiotensinogen (*ATG*) and renin (*REN*) [86]. The researchers generated kidney organoids with genetically disrupted RAAS from gene-edited *ACE*-/- hiPSCs, *AGTR1*-/- hiPSCs and AR-RTD patient-derived hiPSCs [87].

The disruption of *RAAS* genes does not disrupt proximal tubule patterning in hiPSC-derived kidney organoids grown under standard or hypoxic conditions. Organoids transplanted under kidneys of immunodeficient mice reveal the dependence on RAAS and VEGF-A for engraftment. Hypoxia induces VEGF-A expression and rescues engraftment of *AGTR1*-/- organoids. It is concluded that proximal tubule dysgenesis in AR-RTD is primarily a non-autonomous consequence of delayed angiogenesis.

### 3.11. Nephrotic Syndrome

Podocytes possess interdigitating foot processes that are bridged by a protein complex called the slit diaphragm, which contains proteins such as nephrin (*NPHS1*) and podocin (*NPHS2*). Mutations in the *NPHS1* or *NPHS2* gene lead to congenital nephrotic syndrome, resulting in impaired slit diaphragm formation in glomerular podocytes [88,89]. Tanigawa and coworkers generated iPSC-derived organoids from a patient with *NPHS1* missense mutation, identified impaired NEPHRIN localization and slit diaphragm formation in podocytes [90,91]. Jansen and his team generated iPSC-derived organoids from a patient with an *NPHS2* mutation, showing poor NPHS2 expression and aberrant NPHS1 localization, which was reversible after genetic correction [92]. Majmundar et al. modeled an *NOS1AP* patient variant in knock-in human kidney organoids, revealing malformed glomeruli with increased apoptosis [93].

## 4. The Limitation of Kidney Organoids and Future Perspectives

With the significant advantages of organoids over the conventional 2D culture method, interest in kidney organoids has grown in the research for genetic kidney disease. Validating new treatments or drug candidates for genetic kidney diseases in vitro is essential for identifying effective treatments. It is expected that, in the future, the utilization of various genetic kidney disease organoid models introduced thus far will become widespread for validating the efficacy and safety of new drugs. In addition, with the ability to produce organoids reflecting the disease characteristics of patients, personalized therapies considering individual genetic mutations and physiological characteristics will be feasible.

However, they still face critical deficiencies compared to primitive organs [94]. One major limitation is the lack of vasculature in organoids, leading to their restricted growth potential and susceptibility to cell death. Nonetheless, when transplanted under the kidney capsule in mice, the organoids showed signs of growth, integration with the host circulatory system and further maturation, indicating potential pathways for overcoming this limitation [39].

Additionally, the absence of immune cells also limits their use in studying inflammatory responses, which are crucial in many kidney diseases. This limitation underscores the need for organoids to include key components of human physiology to accurately model disease processes. Moreover, kidney organoids generally represent the kidney’s immature form, raising questions about their suitability for modeling diseases that predominantly manifest in adulthood, such as Fabry’s disease, or conditions involving long-term DNA damage and repair deficiencies, like Karyomegalic Interstitial Nephritis (KIN).

Additionally, laboratory-cultured organoids exhibit significant disparities in cellular composition when compared to original organs. Through RNA sequencing analysis conducted by Wu and his team, it was discovered that organoids consist of 10–20% off-target cells. Furthermore, Wu’s findings revealed that the proportion of each renal cell type considerably varies based on the cell line and the organoid culture protocol applied [95]. For example, the podocyte cluster’s percentage was substantially higher using Morizane’s [27] differentiation scheme compared to Takasato’s [24] (28% and 4%, respectively). Therefore, when modeling specific human diseases, there is a need to focus on the efficiency and reproducibility of differentiation protocols that produce specific cell types.

In conclusion, due to these limitations, kidney organoids still often fail to sufficiently represent the morphological, physiological and functional characteristics of genetic renal diseases.

Recent advancements in organoid technology have addressed limitations by integrating vascular structures through co-culture systems and microfluidic devices. For example, Gabbin et al. developed a microfluidic chip that co-cultures heart and kidney organoids, enhancing nutrient delivery, waste removal and overall maturation and functionality [96]. Song et al. further emphasized the benefits of heart–kidney-connected organoids, which mimic in vivo conditions and allow for the study of inter-organ interactions, crucial for understanding diseases like cardiorenal syndrome [97]. This co-culture setup maintains tissue viability and specific functions, providing a dynamic environment that accurately mimics physiological conditions.

One intriguing question is whether it would be possible to create organoids that produce urine. Specifically, how would the necessary blood pressure for primary urine production be generated? Integrating advanced microfluidic systems and vascular structures could potentially recreate this blood pressure within organoids. The co-culture systems and microfluidic platforms enhance the development of organoids with more physiologically relevant characteristics. These advancements make it feasible to approach the complexities involved in urine production within a laboratory setting, providing a promising direction for future research.

Additionally, high-throughput screening platforms using kidney organoids have accelerated the discovery of new therapeutic compounds [14,35,98,99,100]. For example, in ADPKD research, kidney organoids have been used to identify potential drugs targeting specific signaling pathways involved in cyst formation. This approach provides a deeper understanding of disease mechanisms and facilitates the development of personalized treatment strategies based on the genetic profile of individual patients.

Future improvements in organoid technology, such as enhanced cell maturation through improved culture conditions or the introduction of microfluidic systems, may enable a closer approximation of the mature kidney state for more accurate disease modeling. Efforts to incorporate vascular and immune cell components into organoids could further address current limitations, providing a more representative and functional model for studying kidney diseases and evaluating therapeutic interventions. Recently, improved cell culture with 3D-bioprinting or the microfabrication technique has enhanced cell maturation and reproducibility [14,101,102]. With the advancement of more sophisticated techniques for producing kidney organoids that closely mimic human kidneys, it is expected that in-depth research on genetic kidney diseases and the development of therapeutic interventions will progress at an accelerated pace.

There is a compelling need to broaden the hiPSC lineage to encompass a broader array of kidney diseases. This will lead to a more detailed understanding of the role of genotype in the disease process, by revealing whether specific disease mechanisms exist in patients with the same diagnosis but different genetic backgrounds. This insight will enable sophisticated and personalized therapeutic approaches for patients with genetic kidney disease.

## 5. Conclusions

Using kidney organoids to investigate the mechanism of genetic kidney diseases helps deepen our understanding of the onset mechanisms of these conditions. It is crucial to examine how genetic mutations affect the development and function of the kidneys and to mimic abnormalities in kidney cells and tissues. Gene editing technologies like CRISPR/Cas9 have facilitated research in this area, enabling the generation of knockout lines and correction of mutations in patient-derived hiPSCs, advancing our understanding of human kidney organogenesis. Future efforts should aim at refining organoid technology to better mimic human kidney complexity, enabling more accurate disease modeling and therapeutic discovery. Overcoming these obstacles will be crucial for utilizing organoids in personalized medicine and advancing genetic kidney disease research.

## Figures and Tables

**Table 1:** Examples for the modeling of genetic kidney disease using kidney organoids.

Disease	Gene	Phenotype and Key Finding	Refs.
Autosomal Dominant Polycystic Kidney Disease	*PKD1 or PKD2*	Cyst formation from tubules Cyst formation from tubules	[26,35,43] [44]
		Cyst formation from Ureteric bud	[45]
	*GANAB, DZIP1L*	Cyst formation from tubules	[46]
Autosomal Recessive Polycystic Kidney Disease	*PKHD1*	Cyst formation from tubules	[47]
Fabry Disease Nephropathy	*GLA*	Deformation of podocytes and tubular cells with accumulation of Gb3, increased oxidative stress and apoptosis	[48]
		Decreased α-Gal A activity and increased Gb3 deposition according to disease severity	[49]
Gitelman Syndrome	*SLC12A3*	Decreased expression of NCCT protein and RNA	[50,51]
Karyomegalic Interstitial Nephritis	*FAN1*	DNA repair impairment	[45,52]
Alport Syndrome	*COL4A5*	Altered expression of Type 4 Collagen α5	[53,54]
APOL1 Nephropathy	*APOL1*	Transcriptomic profiling of *APOL1* mutant organoid	[55,56,57]
Nephronophthisis	*IFT140*	Shortened, club-shaped primary cilia and Polarization defect	[58]

* Gb3, globotriaocylceramide; NCCT, Sodium–chloride cotransporter.
